# Comprehensive diagnostic approaches to feline toxoplasmosis: Bridging traditional methods and emerging technologies

**DOI:** 10.1080/21505594.2025.2563766

**Published:** 2025-09-19

**Authors:** Dan Zhao, Yanzhen Liao, Hao Liu, Jianwei Wang, Ruiying Liang, Rongqiong Zhou, Jiabo Ding, Sixin Zhang, Xinming Tang

**Affiliations:** a College of Veterinary Medicine, Southwest University, Chongqing, China; b Key Laboratory of Animal Biosafety Risk Prevention and Control (North) & Key Laboratory of Veterinary Biological Products and Chemical Drugs of MARA, Institute of Animal Science, Chinese Academy of Agricultural Sciences, Beijing, China

**Keywords:** Toxoplasmosis, life cycle, diagnostic approaches, definitive host, transmission blocking

## Abstract

*Toxoplasma gondii* is a globally distributed intracellular parasite, with felids serving as its definitive hosts and playing a central role in environmental contamination through oocyst shedding. Accurate and timely diagnosis in cats is critical for interrupting transmission cycles and mitigating public health risks. This review provides an integrated overview of current and emerging diagnostic strategies for feline toxoplasmosis, encompassing serological assays, molecular methods, and nanomaterial-enhanced technologies. Comparative analysis highlights the diagnostic performance, advantages, and limitations of each method across diverse settings. The incorporation of artificial intelligence and machine learning is expected to enhance diagnostic precision, enabling stage-specific detection and personalized intervention strategies. Emphasis is placed on the need for standardized diagnostic protocols and the identification of antigens with high expression in schizogony, bradyzoite, and sporulated oocyst stages – key developmental phases relevant to early detection. This review provides valuable insights into the technical bottlenecks that need to be addressed and future development directions for diagnostic methods of feline toxoplasmosis, which holds significant importance for toxoplasmosis prevention and control.

## Introduction

**Figure 1.**
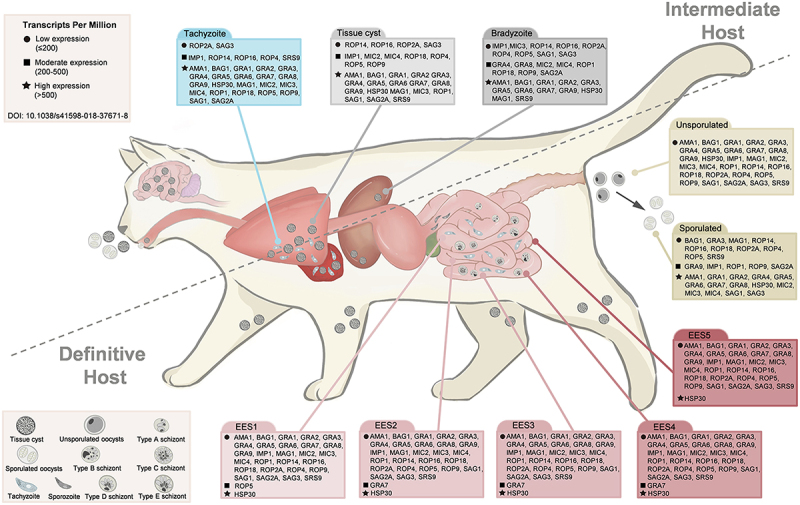
Correlation between *T. gondii* diagnostic antigen expression levels and life cycle stages. the expression levels of diagnostic targets compiled from the literature were compared across different life stages of toxoplasma gondii, and the relationship between antigen expression patterns and their suitability as diagnostic targets was systematically examined. intermediate host: GRAs show high overall expression; ROPs and MICs demonstrate relatively lower expression. definitive host: GRA7 and HSP30 maintain moderate-to-high expression throughout most life cycle stages. life cycle stage-specific expression patterns: tachyzoite stage (blue): systemic dissemination via bloodstream; numerous highly expressed protein types. Tissue cyst stage (gray): abundant cysts in tongue, brain, lungs, heart, spleen, and muscles; multiple highly expressed cyst-specific proteins. Intestinal schizonts (EES1-EES5, red) & unsporulated oocysts (light yellow): predominantly low protein expression. Sporulated oocysts (dark yellow): marked increase in highly expressed proteins. Different shapes represent varying TPM: circle: low expression (TPM ≤200); square: moderate expression (200 < TPM ≤ 500); star: high expression (TPM > 500). All TPM data were obtained from the ToxoDB database website (https://toxodb.org/toxo/app).

Toxoplasmosis, caused by the obligate intracellular protozoan *Toxoplasma gondii*, is one of the most widespread zoonotic infections globally, affecting nearly all warm-blooded animals, including humans. The life cycle of *T. gondii* consists of two key phases: sexual reproduction, which occurs exclusively in feline intestines, and asexual reproduction, taking place in intermediate hosts or feline. When feline ingest tissue cysts or infectious oocysts from infected prey or contaminated water, some parasites differentiate into bradyzoites, forming persistent tissue cysts [1]. Meanwhile, others invade intestinal epithelial cells, undergoing sexual reproduction and producing unsporulated oocysts. These oocysts are excreted and, under favorable environmental conditions, mature into infectious sporulated oocysts [2,3]. Current laboratory diagnosis of *T. gondii* primarily relies on detecting its secretory antigens, whose expression levels vary according to the parasite’s life cycle stage (Figure 1) [4].

Human toxoplasmosis presents in two major clinical forms: congenital and acquired. Congenital infection results from vertical transmission during pregnancy and can lead to severe outcomes, including miscarriage, hydrocephalus, and stillbirth [5]. Acquired toxoplasmosis is typically asymptomatic in immunocompetent individuals but can cause life-threatening complications such as encephalitis, neurological disorders, ocular lesions, and systemic infections in immunocompromised patients [6].

Human infection typically occurs through ingestion of sporulated oocysts from contaminated water, food, or soil, or through consumption of undercooked meat containing tissue cysts [7,8]. Notably, both infectious forms trace back to feline hosts, making cats a critical reservoir for the environmental dissemination of *T. gondii*. This highlights the central role of felids in the parasite’s transmission dynamics and the necessity of targeted surveillance in these populations.

Epidemiological data indicates substantial variation in *T. gondii* prevalence across regions and host species. Domestic cats exhibit a seroprevalence of approximately 35% (7/20), whereas rates in wild felids can exceed 59% (3/5) [9]. Moreover, regional surveys have reported a seroprevalence of 24% (1/4) among cats in mainland China (1991–2015), comparable to that in several European countries, while countries like Japan report significantly lower rates of around 5.4% (3/50) [10]. Kareem Hatam-Nahavandi *et al*. conducted a systematic review and meta-analysis, which revealed that roughly one in every 50 cats is actively shedding oocysts at any given time, contributing to environmental contamination [11].

Given the global distribution of cats and the public health risks associated with toxoplasmosis, improved surveillance and diagnostic measures targeting feline hosts are essential. Early detection and effective prevention strategies can reduce environmental oocyst contamination and mitigate the risk of transmission to both humans and animals.

## Diagnostic methods for T. gondii infection

### Serological tests

#### Enzyme-linked immunosorbent assay (ELISA)

ELISA is a widely used serological technique based on the specific interaction between antibodies and their corresponding antigens. It employs enzyme-labeled secondary antibodies to amplify the detection signal, allowing for highly sensitive and specific results. ELISA is cost-effective, scalable, and particularly valuable for early-stage diagnosis due to its ability to efficiently detect IgM antibodies. It is commonly used in clinical diagnosis of various diseases, including toxoplasmosis.

ELISA is widely used in toxoplasmosis diagnosis, leveraging various recombinant antigens to enhance sensitivity and specificity. Among the most commonly employed antigens are surface antigen 1 (SAG1), dense granule antigen 7 (GRA7), immune-mapped protein 1 (IMP1), and apical (Table 1) [26,29–31]. Critical assay parameters, such as serum dilution, the concentration of HRP-conjugated secondary antibodies, and the composition of the diluent, are fine-tuned to maximize performance. This process effectively reduces nonspecific background signals, enhancing the specificity and reliability of the iELISA method, whose diagnostic value must ultimately be validated through clinical application. To date, numerous recombinant *T. gondii* proteins have been evaluated in ELISA formats, with most demonstrating high sensitivity and specificity [32,33]. However, hemolysis or blood samples containing high fat may lead to false positive or false negative results in case of improper handling. Secondly, if the antibody production is less, the detection efficiency will also become low, and weak positive is also easy to be confused with negative. In addition, subsequent data analysis is also crucial [34].

**Table 1.** Serological diagnostic methods established for different antigenic targets of *T. gondii.*

Target gene	Gene ID	ELISA	ICT	GICA	TRFIA	Reference
SAG1	TGME49_233460	+	–	–	SAG1- GRA8	Qi et al. [12]; Huertas-López et al.[13]
SAG2	TGME49_271050	+	+	–	+	Lau & Fong [14]; Huang et al. [15]; Huertas-López et al.[13]
SAG3	TGME49_308020	+	+	–	–	Motazedian et al. [16]; Luo et al.[17]
GRA1	TGME49_270250	+	–	–	–	Lecordier et al. [18];
GRA3	TGME49_227280	+	–	–	–	Wang et al.[19]
GRA6	TGME49_275440	+	–	–	–	Lecordier et al. [18];
GRA7	TGME49_203310	+	+	–	+	Qi et al. [12]; Ybañez et al. [20]; Huertas-López et al.[13]
GRA8	TGME49_254720	+	–	–	SAG1- GRA8	Jirapattharasate et al. [21]; Huertas-López et al.[13]
ROP1	TGME49_309590	+	–	–	–	Aubert et al.[22]
ROP5	TGME49_308090	+	–	–	–	Grzybowski et al.[23]
ROP14	TGME49_315220	–	+	–	–	Yang et al.[24]
ROP18	TGME49_205250	+	–	–	–	Grzybowski et al.[23]
BAG1	TGME49_259020	+	–	–	–	Qi et al.[12]
MAG1	TGME49_270240	+	–	–	–	Holec et al.[25]
AMA1	TGME49_255260	+	–	+	–	Gao et al. [26]; Fan et al.[27]
MIC3	TGME49_319560	+	–	–	–	Fatima et al.[28]
IMP1	TGME49_293470	+	–	–	–	Dong et al.[29]

“+:” have been established; “-:” have not yet been established or reported.

#### Modified agglutination test (MAT)

MAT is widely recommended as a standard experimental method for the serological diagnosis of toxoplasmosis. Its principle is based on the specific binding of antigens to antibodies present in serum, resulting in the formation of antigen-antibody complexes. When antibodies are present in a sample, they bind to fixed antigens, producing a visible agglutination reaction that can be observed with the naked eye or under a microscope [35].

MAT is highly sensitive and capable of detecting low concentrations of antibodies, making it suitable for identifying early-stage infections. The procedure is relatively simple, rapid, and practical for clinical use. Additionally, the extent of agglutination enables semi-quantitative analysis, allowing for the estimation of antibody levels. Comparative studies have shown that the sensitivity and specificity of MAT for detecting *T. gondii* antibodies in cat serum are comparable to those of the Indirect Fluorescent Antibody Test (IFAT) [36]. MAT has also demonstrated excellent diagnostic performance across various hosts. However, like all serological methods, MAT has certain limitations. False-positive or false-negative results may occur, often influenced by factors such as sample handling, processing, and storage conditions. Moreover, while MAT can confirm the presence of antibodies against *T. gondii*, it does not differentiate between recent and past infections, nor does it identify the specific stage of infection. Therefore, confirmatory testing using reference samples or complementary diagnostic methods is often necessary [37].

#### Indirect fluorescent antibody test (IFAT)

IFAT is a widely used method for detecting *T. gondii* gondii-specific antibodies in feline serum. In this technique, feline serum is incubated with *T. gondii* antigens, allowing any specific antibodies present to bind to the antigens. A fluorescently labeled anti-feline secondary antibody is then added, which binds to the primary antibodies that have attached to the antigens. When these immune complexes form, the fluorescence can be visualized under a fluorescence microscope [38].

IFAT is valued for its high sensitivity and specificity, enabling both the direct observation of antigen – antibody interactions and semi-quantitative analysis based on fluorescence intensity. However, the procedure is technically demanding, requires meticulous handling, and is prone to variability. False-negative or false-positive results may occur, particularly when technical execution or result interpretation is suboptimal. Studies have shown that the sensitivity and specificity of IFAT can be significantly influenced by several factors, including the skill level and experience of laboratory personnel, as well as the precision of fluorescence interpretation. These limitations affect the consistency and reproducibility of IFAT results, making the test less suitable for routine use in all laboratories and necessitating trained technicians for reliable performance [39]. Compared to other methods, SAG1-based ELISA has shown excellent diagnostic performance in detecting *T. gondii* antibodies in feline. This approach offers a simpler protocol, easier standardization, and high sensitivity, making it more practical than IFAT for large-scale screening and widespread applications [40]. Although IFAT is no longer the primary screening method, it remains the gold standard for *T. gondii* detection and retains unique value in specialized applications. When used in combination with ELISA, it can further improve diagnostic efficiency [41,42]. Using ELISA for preliminary screening of *T. gondii* infection, followed by secondary verification with IFAT, not only optimizes the detection process and improves efficiency, but also effectively reduces the overall cost of detection [43].

#### Immunochromatographic tests (ICT)

ICT rely on the principle of antigen – antibody binding and offer the advantages of rapid, convenient pathogen detection without the need for sophisticated equipment. These tests typically require only a few drops of sample and can produce results within approximately 30 minutes, making them well-suited for point-of-care or field diagnostics. However, due to insufficient antibody production in early infection, the sample is easily judged as false negative, and this method is only applicable for qualitative analysis [33].

In the context of *T. gondii* detection in cats, ICT is commonly used to identify specific antigens or antibodies (usually IgM or IgG) against *T. gondii* [44]. Antigens present in blood, feces, or other samples bind to specific antibodies embedded in the test kit, leading to a visible color change on the chromatographic strip. Commercial ICT kits, such as FASTest® TOXOPLASMA g, have been evaluated against established methods like in-house IFAT. Studies show that the diagnostic performance of these rapid tests, particularly in terms of sensitivity and specificity, is comparable to traditional serological methods, demonstrating their reliability [45]. ICT is not only applicable to *T. gondii* detection in cats but is also increasingly used for diagnosing parasitic infections in other animals. For example, SAG3, a surface protein widely used as an ELISA antigen, has shown great potential in ICT applications for pigs [17]. Rhoptry Protein 14 (ROP14) has also been explored for similar purposes [24]. Notably, Ybañez *et al*. were the first to utilize GRA7 as an antigen for feline ICT, testing 100 cats and comparing results with multi-antigen iELISA [20,46]. Their findings demonstrated strong agreement between the two methods, confirming the test’s reliability. In addition, surface SAG2—a key molecule involved in *T. gondii* host cell invasion – is currently being used as an antigen in rapid ICT assays for detecting anti-*T. gondii* antibodies in cats (Table 1) [14,15]. Looking ahead, advances in nanotechnology and the development of multiplex ICT assays incorporating multiple *T. gondii* antigens may further enhance the sensitivity and diagnostic accuracy of this method, providing more powerful tools for the detection of toxoplasmosis in both veterinary and public health settings.

#### Gold immunochromatographic assay strips (GICA)

GICA is a type of immunochromatographic technique that utilizes gold nanoparticles to label antibodies or antigens. These nanoparticles exhibit distinct colorimetric changes, allowing results to be observed directly with the naked eye as they migrate along the test strip. The assay delivers results within minutes, making it a rapid and convenient diagnostic tool, especially in settings lacking specialized laboratory equipment. Compared to ELISA, GICA offers a faster turnaround time and simpler operation. In recent research, GICA was developed using *T. gondii* AMA-1 and was evaluated against a commercially available ELISA that also used AMA-1. The GICA method demonstrated higher sensitivity and specificity. In terms of antigen selection, AMA-1-based GICA outperformed tests using GRA7, indicating superior diagnostic performance (Table 1). Additionally, the AMA1C-GICA platform has been integrated with digital evaluation technologies, enhancing accuracy by minimizing human error and enabling more precise quantitative analysis [27].

#### Time-resolved fluorescence immunoassay (TRFIA)

**Figure 2.**
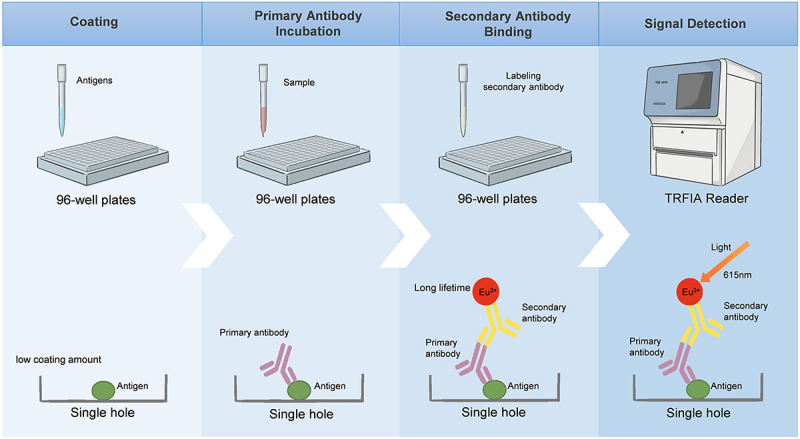
Flowchart and advantages of TRFIA for detection of *T. gondii* specific IgG/IgM Antibodies. The key steps of this procedure are as follows: Coating: *T. gondii* antigens are immobilized in 96-well plates; Binding: serum antibodies from test samples bind to the coated antigens; detection: antigen-antibody complexes are formed with Eu^3 +^ -labeled secondary antibodies; measurement: a TRFIA instrument excites the Eu^3 +^ label at 340 nm, and fluorescence intensity is measured at 615 nm. Advantages of TRFIA: high sensitivity: capable of detecting even low-concentration samples; reduced background interference: the long fluorescence lifetime of Eu^3 +^ allows delayed measurement, minimizing autofluorescence and enhancing specificity and accuracy; excellent stability: Eu^3 +^ -labeled compounds are highly stable, making them suitable for long-term storage and repeated use.

TRFIA is a highly sensitive technique that integrates time-resolved fluorescence detection with immunoassay principles. It utilizes fluorescent markers, such as the lanthanide ion europium, which possess a long fluorescence lifetime and extended signal duration. During detection, the instrument first excites all fluorescent substances to allow background fluorescence to decay, and then reads the remaining fluorescent signals. At this point, the signals are almost entirely from the lanthanide markers on the target substances, which allows TRFIA to significantly reduce background fluorescence interference, resulting in clearer and more accurate measurements of antigens or antibodies in a sample (Figure 2). Ana Huertas-López *et al*. developed multiple TRFIA methods using recombinant antigens of *T. gondii*, including GRA7, truncated GRA7, SAG2, truncated SAG2, and chimeric antigens of SAG1 and GRA8. The performance of these TRFIAs was evaluated by comparing them with commercial ELISA and MAT methods. The results showed that the TRFIA method based on SAG1-GRA8 chimeric antigen exhibited better detection performance than a single antigen in detecting *T. gondii* infection in feline. Compared to assays employing single recombinant antigens, TRFIA with chimeric antigens showed enhanced sensitivity and specificity [13] (Figure 2). These findings suggest that chimeric antigens may be advantageous for improving diagnostic accuracy in other immunoassays, including GRA7- or SAG2-based ELISAs and GRA7-based ICTs (Table 1) [20,47].

### Antigen and molecular-based diagnosis

#### Polymerase chain reaction (PCR) and advanced PCR

PCR is a molecular technique that enables the in vitro amplification of specific DNA sequences. In the diagnosis of *T. gondii* infection, DNA is typically extracted from the blood, tissues, or other biological samples of cats. Specific primers targeting conserved *T. gondii* genes, such as the B1 gene and REP-529 repeat element, are used to amplify the target DNA through repeated cycles of denaturation, annealing, and extension [48]. The resulting amplification products are then detected using gel electrophoresis or fluorescent dyes to confirm the presence of *T. gondii*-specific DNA. PCR offers high sensitivity and specificity. The use of gene-specific primers enhances diagnostic accuracy and reduces the likelihood of false-positive results. Moreover, PCR can differentiate between *T. gondii* strains and provides rapid, precise detection, particularly valuable when serological testing is inconclusive [49]. For example, during the initial phase of *T. gondii* infection, specific antibody levels in feline serum may remain low or fall below the detection threshold. Under such circumstances, PCR-based detection of tissue samples often provides more reliable diagnostic results [50].

Advanced PCR techniques such as real-time quantitative PCR (qPCR) and nested PCR are commonly employed for improved sensitivity and quantification [51–53]. Studies have demonstrated that qPCR often outperforms traditional serological methods such as the MAT, especially in postmortem tissue analysis [54]. One of the key advantages of qPCR is its ability to distinguish *T. gondii* from closely related species, such as *Hammondia hammondi*. Unlike *T. gondii, H. hammondi* does not form tissue cysts, making this differentiation critical for accurate diagnosis and epidemiological studies. The high specificity of qPCR-based detection enhances diagnostic confidence, particularly in tissue samples. In recent years, the qPCR detection system based on recombinant proteins such as SAG1, MAG1 and BAG1 has further expanded the application range of this technology [55–57]. However, qPCR requires specialized laboratory equipment and trained personnel, which may limit its use in routine clinical or field settings. Nested PCR has a significant sensitivity advantage over conventional PCR and can effectively detect clinical samples with very low pathogen load. However, this technology has limitations such as complex operation process, long time consumption and high detection cost. At present, nested PCR has been widely used in the detection of a variety of *T. gondii* specific targets, including B1 gene, ITS-1 sequence, GRA6 gene and GRA7 gene, etc (Tables 2 and 3) [52,66,73,81]. Although many detection methods are superior to PCR in sensitivity, PCR remains an essential tool for confirming *T. gondii* infection and characterizing parasite strains with high precision. Conventional PCR methods typically require specialized instruments and highly trained personnel. These factors limit the application of PCR in on-site diagnostics.

**Table 2.** Gene-targeted etiological diagnostic assays for *T. gondii.*

Target gene	Gene ID	PCR or advanced PCR*	LAMP	CRISPR/Cas	Reference
B1	/	q, n+	+	+	Useche et al. [58]; Belaz et al. [51]; Zamora-Vélez et al. [52]; Karakavuk et al. [59]; Lei et al.[60]
REP 529	/	q+	+	+	Gorgani-Firouzjaee et al. [61]; Belaz et al. [51]; Karakavuk et al. [59]; Ma et al.[62]
SAG1	TGME49_233460	q+	+	–	Jones et al. [63]; Yu et al. [55]; Lau et al.[64]
SAG2	TGME49_271050	n+	+	–	Yücesan [65]; Fazel et al. [66]; Lau et al.[64]
SAG3	TGME49_308020	n+	–	–	Sudan et al. [67]; Rather et al.[68]
BAG1	TGME49_259020	q+	–	–	Paredes-Santos et al. [69]; Enshaeieh et al.[70]
GRA6	TGME49_275440	n+	–	–	Nora et al. [71]; Fazel et al.[66]
GRA7	TGME49_203310	n+	–	–	Zheng et al. [72]; Costa et al.[73]
ROP5	TGME49_308090	+	–	+	Zhao et al. [74]; Behnke et al.[75]
ROP18	TGME49_205250	n+	–	+	Zamora-Vélez et al. [52]; Azimpour-Ardakan et al. [76]; Kong et al.[77]
MAG1	TGME49_270240	q, n+	–	–	Zhuo et al. [78]; Khanaliha et al.[56]
ITS-1	/	q, n+	+	-^†^	Cong et al. [79]; Rahumatullah et al. [80]; Burrells et al. [81]; Zhuo et al.[82]
18S rDNA	/	n+	+	–	Jones et al. [63]; Mikita et al.[83]

*.Advanced PCR including qPCR and nested PCR.

†.“+:” have been established; “-:” have not yet been established or reported; “/:” non-coding genes.

**Table 3.** Comparative evaluation of etiological diagnostic methods for *T. gondii* infection.

Method	First Introduced	Target Genes	Sensitivity	Specificity	Speed	Equipment Required	Cost	Ease of Use	Field Applicability	Current Application Status
PCR	Widely established	B1, RE 529-bp	High	High	Moderate	Thermal cycler	Low	Requires trained personnel	Limited	Routine diagnostic tool
qPCR (Real-Time PCR)	Widely established	B1, REP-529	High	Very High	Fast	Real-time PCR	High	Requires trained personnel	Good	Clinical standard
Nested PCR	Later than PCR	B1, SAG1	Very High	High	Moderate	Two-step PCR	Medium	Moderate	Limited	Research, low-load samples
LAMP	Later than PCR	B1	Very high	High	Fast	Water bath or heat block	Low-Medium	User-friendly	Good	Increasing use in field diagnostics
CRISPR/Cas9	Most recent (experimental)	RE 529-bp (with RPA)	Extremely high	Very high	Very fast	Minimal (isothermal + lateral flow)	Potentially low	Simplified (under development)	Excellent (in progress)	Experimental, under development

#### Loop-mediated isothermal amplification (LAMP)

LAMP reactions are carried out under constant temperature conditions (typically 60–65°C) using four to six specially designed primers and a strand-displacing DNA polymerase, such as Bst DNA polymerase [84]. The amplification process produces a large amount of DNA in a short time, and results can be directly visualized via fluorescent dyes or by observing a white precipitate of magnesium pyrophosphate. Alternatively, turbidity changes can be measured photometrically [85].

Previous studies have successfully applied LAMP to *T. gondii* detection. For example, one approach targeted the highly repetitive 529-bp sequence of *T. gondii*, using primers labeled with fluorescein isothiocyanate to enable detection. Amplification products were visualized using lateral flow dipsticks (LFDs), resulting in a LAMP-LFD assay with excellent specificity and sensitivity. A notable limitation of this method is its ability to detect only early-stage infections, as the detection relies on the presence of circulating parasite DNA [86]. However, recent studies have successfully integrated recombinant proteins (e.g. SAG1 and SAG2) with LAMP technology, demonstrating high sensitivity and specificity. This innovative approach offers a promising new strategy for accurate *T. gondii* infection detection [64]. In another study, researchers developed a colorimetric LAMP-RE assay targeting the *T. gondii* B1 gene, which allowed for the detection of parasite DNA in cat feces. This adaptation of the LAMP method further demonstrates its versatility and potential in field diagnostics (Tables 2 and 3) [59]. Overall, LAMP represents a promising tool for the rapid, on-site detection of toxoplasmosis in cats, especially in resource-limited settings where access to advanced diagnostic infrastructure is restricted.

#### RPA-CRISPR/Cas9 based methods

CRISPR/Cas9 is a powerful gene-editing technology derived from the adaptive immune system of bacteria. It consists of a Cas9 endonuclease and a guide RNA (gRNA), which directs Cas9 to a specific DNA sequence for precise recognition and cleavage [87]. The CRISPR-Cas9 system facilitates precise genetic modifications, enabling targeted knockout of virulence-associated genes (e.g. ROP5 or ROP18) to investigate genotype-dependent virulence variations in *T. gondii* [75]. Furthermore, this technology shows growing potential for molecular diagnostic applications. In the context of *T. gondii* detection, a novel diagnostic method was developed by combining recombinase polymerase amplification (RPA) with CRISPR/Cas9 technology to detect *T. gondii* DNA in feline fecal samples. This approach targets the highly repetitive 529-bp RE fragment of *T. gondii*, and detection results are visualized using lateral flow strips [88]. Compared with conventional PCR, RPA does not require thermal cycling [89,90]. When integrated with the highly specific DNA cleavage activity of the CRISPR/Cas9 system, this method provides a rapid, sensitive, and field-deployable alternative for detecting *T. gondii*. The combined RPA – CRISPR/Cas9 platform holds significant potential for practical applications, particularly in low-resource settings or for point-of-care diagnostics in veterinary medicine (Tables 2 and 3).

## Nanomaterial encapsulation in diagnosis and application

Biodegradable copolymers such as poly (lactic-co-glycolic acid) (PLGA), composed of polylactic acid and polyglycolic acid, have shown considerable potential as delivery systems for drugs and vaccines targeting *T. gondii*. Several studies have successfully encapsulated recombinant SAG-1 into PLGA nanoparticles, demonstrating that intranasal administration can elicit a robust humoral immune response [91]. Among the various antigens tested, rSAG1 remains the most extensively studied in PLGA-based vaccine formulations. Furthermore, PLGA nanoparticles encapsulating the rhoptry protein ROP18 have been shown to induce significant protective immunity following immunization [92]. In another study, a DNA vaccine encoding *T. gondii* oxidoreductase delivered using a chitosan-PLGA nanoparticle formulation induced both humoral and cellular immune responses in mice, resulting in the production of high titers of specific antibodies and contributing to effective prevention and control of infection [93].

Nanomaterials have also been explored for diagnostic applications. A detection platform utilizing carbon nanofibers and gold nanoparticles has demonstrated high sensitivity in identifying anti-*T. gondii* IgG antibodies. Gold nanoparticles enhance the electrochemical signal of biosensors, facilitate the immobilization of *T. gondii* antigens on the sensor surface, and enable the formation of antigen – antibody complexes, thereby allowing accurate serological detection of *T. gondii* infections [94].

## Evaluation of diagnostic efficacy

### Comparative studies of diagnostic tests

Among serological diagnostic methods for *T. gondii* infection, the IFAT requires collecting and transporting feline blood samples to a laboratory equipped with specialized testing instruments and trained personnel. Consequently, this method is unsuitable for field-based applications [95,96]. The ELISA produces a colorimetric response, and in cases of strong seropositivity, results may be visually interpreted on site for qualitative assessment [97]. However, quantitative interpretation still requires laboratory-based analysis. ICTs operate on a lateral flow principle, where a visible color change on the test strip facilitates rapid, on-site detection [98]. Similarly, the MAT is applicable in field settings, as positive results yield a characteristic agglutination pattern that can be observed with the naked eye or under low-magnification microscopy. In contrast, molecular diagnostic methods such as PCR require sample processing (e.g. feces, blood, or tissue) followed by nucleic acid extraction and amplification using laboratory equipment. Although LAMP offers operational simplicity and faster turnaround under isothermal conditions, it remains constrained to laboratory environments due to equipment and technical requirements. Likewise, CRISPR/Cas9-based detection, while offering enhanced specificity and sensitivity, is currently limited to research and laboratory settings and is not yet feasible for on-site diagnostics.

### Case studies from various geographical regions

ELISA and PCR were employed to assess *T. gondii* infection in 197 domestic and stray cats across multiple regions of Khyber Pakhtunkhwa Province, Pakistan. The results indicated a significantly higher infection rate in stray cats compared to domestic ones, likely due to the increased exposure stray cats to complex and unsanitary environments. Additionally, older cats exhibited a higher prevalence of infection, often presenting chronic clinical symptoms [99]. These findings suggest that targeted screening programs should prioritize high-risk populations, particularly stray and senior cats, to facilitate early detection and timely intervention in the infection and transmission of *T. gondii*.

In Colombia, PCR analysis of feline fecal samples revealed a strong association between urban stray cat populations and the high prevalence of *T. gondii* in the human population, emphasizing the zoonotic risk posed by unmanaged cat populations [52]. Similarly, a survey conducted in the central-northern region of Oklahoma, USA, found that approximately 63.9% of feral cats were infected with at least one parasitic species, highlighting the substantial burden of parasitic infections among wild feline populations [100].

In Malaysia, fecal sample analysis using PCR targeting the B1 and REP genes demonstrated significantly enhanced sensitivity and diagnostic accuracy [101]. These multicopy-targeted PCR methods have shown considerable promise for detecting *T. gondii*, outperforming single-copy target approaches in terms of both sensitivity and reliability.

While serological assays offer advantages for large-scale epidemiological surveys, PCR methods allow for greater sample diversity – including feces, tissues, and blood – and broader diagnostic applications. Furthermore, multicopy-target PCR targeting genes such as B1 or REP markedly improve the accuracy of detecting *T. gondii* infection. Therefore, integrating multiple diagnostic approaches – combining serological screening with molecular confirmation – can provide a more comprehensive and precise evaluation of *T. gondii* infection in various settings.

### Impact of diagnostic tools on disease control

Serological testing remains one of the most widely employed methods for detecting *T. gondii* infection in cats, primarily through the identification of specific IgG and IgM antibodies in the early stages of infection [102]. Molecular techniques such as PCR can further support diagnosis by detecting *T. gondii* DNA in blood, feces, or tissue samples from suspected cases [103]. As the definitive host, domestic and feral cats play a central role in the environmental dissemination of infectious oocysts. Routine screening of cat populations, coupled with the isolation and treatment of infected individuals, can significantly reduce oocyst shedding. This, in turn, limits transmission to other animals and humans via contaminated air, water, or food [104].

*T. gondii* poses a serious threat to immunocompromised individuals and pregnant women. Therefore, the early diagnosis and management of feline populations are critical public health measures. Special attention should be given to cat owners and individuals in frequent contact with cat feces. In high-density environments – such as breeding facilities, shelters, and farms – monitoring and controlling *T. gondii* infection in cats is particularly important to prevent zoonotic spread and cross-species transmission.

Currently, no effective commercial vaccine exists for the prevention of *T. gondii* infection in cats. However, research has demonstrated that intranasal immunization with rhoptry proteins can reduce oocyst shedding [105]. In particular, recombinant ROP2 (rROP2) protein has shown promising immunogenicity and protective effects [106]. Nevertheless, further studies are required to evaluate the long-term efficacy and safety of such vaccines in real-world settings. Given the absence of effective vaccination, early and accurate diagnosis is essential for identifying and treating infected cats. Timely therapeutic intervention can interrupt the parasite’s life cycle, shorten the period of oocyst excretion, and ultimately contribute to the effective control of *T. gondii* transmission, which is essential for preventing severe toxoplasmosis in pregnant women and immunocompromised individuals and ultimately mitigating the global public health burden of *T. gondii*.

## Novel approaches in diagnostic research

### Integration of advanced technologies

*T. gondii* is characterized by subtle and difficult-to-identify imaging features, which makes the traditional microscopic examination of tissue samples highly dependent on the experiential interpretation of experts. This not only limits diagnostic efficiency but also makes it extremely prone to missed diagnoses or misdiagnoses. Recent advances in Artificial Intelligence (AI) and Machine Learning (ML) offer promising tools to enhance the accuracy, efficiency, and speed of feline toxoplasmosis diagnosis. AI systems can analyze microscopic images of blood smears and tissue sections, automatically detecting morphological indicators of *T. gondii* infection with high precision [107]. For instance, computer vision models trained through transfer learning techniques have demonstrated the ability to identify specific forms of *T. gondii* in clinical samples and detect early-stage infections [108]. These systems significantly reduce the manual burden on laboratory personnel and minimize false positives caused by human error.

In addition to image-based diagnostics, AI enables comprehensive analysis of multiple clinical and biological parameters – including pathogen virulence and host immune responses – to inform both diagnostic and therapeutic strategies. Machine learning algorithms, which identify patterns and make data-driven predictions, can process large datasets encompassing laboratory test results and clinical symptoms [109–111]. This capability supports the development of predictive models that can identify cats at higher risk of *T. gondii* infection, thereby facilitating early intervention and targeted prevention [112,113]. Moreover, AI technologies can foster interdisciplinary collaboration through enhanced data sharing and integration across veterinary, diagnostic, and research domains. This networked approach further increases the efficiency of diagnostic workflows and supports the development of robust, scalable solutions for the control of *T. gondii* infections.

### Development of comprehensive diagnostic panels

Relying on a single diagnostic method carries the risk of false positives or false negatives. Integrating multiple detection techniques can effectively mitigate these limitations and significantly enhance diagnostic accuracy and reliability. Restriction Fragment Length Polymorphism and Sanger Sequencing can effectively distinguish the genotype of *T. gondii* infections, enabling precise diagnosis [114]. Serological assays, such as ELISA and the MAT, are widely used due to their ease of use, rapid turnaround, and suitability for large-scale preliminary screening. Some of these tests allow for on-site interpretation, minimizing errors associated with sample transport and storage [115,116]. ICTs offer additional advantages by providing rapid, user-friendly, and equipment-free preliminary results. For samples that yield positive or ambiguous results in preliminary screenings, molecular methods such as PCR are employed to confirm the presence of *T. gondii* DNA. This two-tiered approach ensures high diagnostic specificity and sensitivity.

The integration of serological, molecular, and immunochromatographic methods not only improves overall diagnostic performance but also increases operational flexibility. Depending on specific circumstances and diagnostic requirements, practitioners can tailor test combinations to optimize efficiency and accuracy while maintaining optimal cost-effectiveness. This multi-method strategy provides robust technical support for early diagnosis, prevention, and control of feline toxoplasmosis.

### Standardization of diagnostic protocols

Establishing unified diagnostic guidelines for *T. gondii* infection in cats is essential for improving consistency in screening, diagnosis, and monitoring. These protocols should be applicable to both domestic and wild cats across diverse environments. Initial screening should begin with a clinical assessment, considering the cat’s symptoms and history of potential exposure. In asymptomatic cats with possible exposure to infection sources, serological testing is recommended as a primary screening method [117,118]. If serological results are inconclusive, molecular methods such as PCR should be employed to confirm infection status [119,120]. Given that PCR testing requires specialized laboratory conditions, strict protocols must be established for sample collection, handling, transportation, and storage to ensure diagnostic accuracy. Standardized operating procedures should be implemented to maintain sample integrity and reliability of test results.

Diagnostic interpretation should be based on a comprehensive evaluation using both serological and molecular methods. To minimize inter-laboratory variability and reduce diagnostic errors, standardized criteria for result interpretation must be developed and uniformly applied across regions. Once infection is confirmed, treatment decisions should be guided by a combination of clinical presentation, infection severity, and the animal’s overall health status. Symptomatic cats should receive timely treatment to mitigate disease progression and reduce the risk of transmission. In contrast, asymptomatic cats may be managed through regular monitoring to track changes in infection status.

Routine epidemiological surveillance is critical to assess regional prevalence and identify high-risk populations. Wild cats, due to their wide-ranging behavior and increased exposure to environmental sources of *T. gondii*, require more stringent monitoring. In endemic areas, regular antibody screening of wildcat populations is recommended. To support long-term monitoring and research, a centralized database should be established to catalog detailed information on *T. gondii*-infected cats, including demographic data, infection status, treatment history, and therapeutic outcomes. This standardized data repository would facilitate coordinated management, enhance data sharing across institutions, and support ongoing research and public health initiatives related to toxoplasmosis.

## Perspective

Currently, most diagnostic methods for *T. gondii* rely heavily on laboratory infrastructure and trained personnel, limiting their applicability across diverse fields and clinical settings. To address this gap, there is a pressing need to develop rapid, user-friendly diagnostic tools that are adaptable to a variety of environments, including low-resource and on-site conditions.

Existing diagnostic approaches also face persistent challenges, including significant cross-reactivity and limited detection efficiency, which remain inadequately resolved. To improve specificity, future efforts should focus on identifying highly specific antigens associated with early-stage infection or employing multi-antigen target strategies to enhance diagnostic accuracy [121,122]. Looking forward, the integration of molecular and serological techniques, complemented by AI, holds great promise for advancing diagnostic precision. AI-assisted analysis can infer the developmental stage of *T. gondii* infection in feline hosts, enabling tailored treatment strategies and improving diagnostic and therapeutic efficiency.

Recent studies on *T. gondii* antigen proteins have revealed that many of these proteins exhibit consistent expression during the middle and late schizogony stages. This observation suggests two key points: first, schizogony in *T. gondii* may be asynchronous, with multiple developmental stages coexisting at a given time point; second, the overall expression levels of these genes during schizogony are low compared to other life cycle stages. These findings may reflect limitations inherent to current experimental models – such as mice and other intermediate hosts – which primarily allow in vivo study of the tachyzoite, bradyzoite, and tissue cyst stages, while providing limited insight into genes expressed during schizogony [4].

For early detection of *T. gondii* infection in cats, the schizogony, sporulated oocyst, and bradyzoite stages represent the most critical developmental phases. To develop serological assays with high sensitivity and specificity, diagnostic targets should prioritize genes that are strongly expressed during these stages. While several high-expression proteins have been identified for the bradyzoite and sporulated oocyst stages, few specific proteins associated with schizogony have been characterized, and those that have are generally expressed at low levels – warranting further investigation.

## Data Availability

Data sharing is not applicable to this article as no new data were created or analyzed in this study.
